# Exploring healthcare provider retention in a rural and frontier community in Northern Idaho

**DOI:** 10.1186/s12913-024-10807-5

**Published:** 2024-03-27

**Authors:** Jonathan D. Moore, Allie M. Lords, Madeline P. Casanova, Ashley J. Reeves, Ann Lima, Cody Wilkinson, Sarah M. Deming, Russell T. Baker

**Affiliations:** 1 WWAMI Medical Education Program, University of Idaho, 875 Perimeter Drive, 83844 Moscow, ID USA; 2 Idaho Office of Underserved and Rural Medical Research, University of Idaho, Moscow, ID USA; 3 St. Mary’s Health & Clearwater Valley Health, Orofino, ID USA; 4 Department of Family Medicine, University of Washington, Seattle, WA USA

**Keywords:** Frontier, NCAQ, Retention, Rural

## Abstract

**Background:**

A shortage of healthcare providers, particularly in primary care and mental health, exists in the predominately rural state of Idaho. There are also barriers to retaining healthcare providers to work in rural and remote communities. Limited research using U.S. samples has explored factors that may affect the retention of healthcare providers in rural areas. Additionally, due to differences between communities, it is important to conduct community-level investigations to better understand how these factors may affect retention in rural areas. Therefore, the purpose of this study was to explore factors affecting healthcare provider retention in a rural community in Northern Idaho.

**Methods:**

A modified version of the Nursing Community Apgar Questionnaire (NCAQ) was completed by 30 healthcare providers in a rural and frontier community in Northern Idaho to assess factors influencing healthcare provider retention. Factors were classified into classes including *geographic, economic, scope of practice*, *medical support*, and *facility and community support classes*. Retention factors were assessed on their perceived importance to retention as well as whether they were perceived as an advantage or challenge to retention based on Likert scales. A “Community Apgar” score was also created by combining the importance and advantage/challenge factors.

**Results:**

Overall, items in the *medical support* group had the highest importance of any other class and included factors such as nursing workforce. Additionally, the *facility and community support* class, which included factors such as televideo support, was rated the highest advantage class and had the highest Apgar score, indicating it contained the factor that healthcare providers identified as the most important advantage (i.e., medical reference resources).

**Conclusion:**

Our study identified multiple factors that healthcare providers deemed as important advantages or disadvantages to retaining healthcare providers in rural areas. Overall, *facility and community support* factors were found to have the highest advantage in the retention of rural providers. Rural healthcare organizations looking to increase healthcare provider retention should target retention efforts towards these factors. Additional research should also be conducted on other rural samples across the U.S. to make comparisons of findings.

## Introduction

An estimated 60 million people live in rural areas in the United States (U.S.) [1]. Additionally, nearly 40% of rural residents live in Health Professional Shortage Areas (HPSAs), making them some of the most underserved populations in the U.S. Individuals who live in rural areas have been reported to be less healthy and have higher rates of death as compared to urban residents [2]. Although the challenge of healthcare equity is multifaceted, the shortage of physicians practicing in rural areas is both a crucial component of the problem, and an avenue of potential remediation [3]. According to the Agency for Healthcare Research and Quality, only 9% of U.S. physicians practice in rural communities [3]. Idaho, a state with a 28% rural population [4], has one of the lowest physicians per capita rates in the U.S. [5]. According to a 2021 report by the Association of American Medical Colleges, Idaho only had 196 physicians per 100,000 residents compared to the overall U.S. rate of 286.5 physicians per 100,000 residents [6]. There is also a shortage of health care providers in Idaho, with 98.7% of counties in Idaho being designated as HPSAs for primary care, and 100% designated as HPSAs for mental health [7]. A report by the Idaho Legislature found that the state needs to increase healthcare staff by 13% (3,000 workers) in order to meet overall U.S. staffing levels [8]. Retention of healthcare professionals in rural and remote communities such as in Idaho is a global health policy concern [9]. 

Providers face a number of unique challenges when practicing medicine in rural areas. For example, a study of mental health providers in Nebraska reported that the burden of paperwork and low reimbursement of Medicaid claims might affect the retention of providers in rural areas, where Medicaid claims are disproportionately high [10, 11]. Additionally, due to the low number of providers per capita in rural areas, professional isolation has been reported among physicians, nurses, and allied health professionals [12]. Researchers have reported that rural physicians work more hours per week than urban physicians, are more likely to be on-call, and have more hospital responsibilities [13]. Physicians in rural areas may also have more difficulty retaining patients’ privacy than rural physicians due to working in smaller towns [14]. 

Multiple factors have been identified as potential barriers or facilitators to retaining physicians in rural areas, including both professional and personal factors [15–17]. A review by Cosgrave et al. identified several themes influencing rural health workforce retention: fulfillment of life aspirations, social connection and place integration, rural familiarity and/or interest, and community participation and satisfaction [17]. Previous research also has reported that factors such as use of telemedicine [18], availability of necessary materials and equipment [19], emergency care [19], nursing workforce [20], and income [21] are important factors affecting provider retention in rural areas.

Furthermore, retaining healthcare providers in rural areas involves consideration of various community-level factors. These include ample employment opportunities for partners [22], education programs for children [23], and a strong sense of community [24, 25]. Researchers studying providers in Australia found the factors perceived to be the most important by physicians were children’s education and partner’s occupation [22]. Additionally, a qualitative study with general practice physicians highlighted the significance of children’s schooling and the impact of professional isolation on the comfort level of practitioners to stay and practice in rural settings [23]. Lastly, community cohesion, particularly strong peer support for providers, has been identified as a crucial factor influencing the retention of healthcare professionals in rural areas [24, 25]. Additionally, researchers have reported long-term retention of rural physicians differed based on participation in either a rural-specific medical education program or standard medical education program; 70.3% of physicians from the rural medical program continued practicing in the same rural area, compared to 46.2% from the standard medical program [26]. Strategies have also been developed and implemented to facilitate provider retention in rural areas [27]. A study on general surgeons in rural areas reported that effective retention was dependent on administrative support, reasonable call and leave schedules, competitive salary, and adequate case variety and volume [27]. Researchers in Norway created a framework for the recruitment and retention of remote rural healthcare workers called the Framework for Remote Rural Workforce Stability [28]. Key elements to this framework included supporting team cohesion, ensuring relevant professional development, and training future professionals [28]. 

Although some factors influencing healthcare provider retention in rural areas have been identified by previous research, findings may vary depending on what rural regions or communities are being studied. Thus, there is a need to examine community-level data to better understand and facilitate health provider retention, specifically as they relate to a rural and frontier state like Idaho. Therefore, the purpose of this study was to explore perceived factors affecting healthcare provider retention in a rural community in Northern Idaho.

## Methods

A survey to evaluate the factors influencing the retention of healthcare providers was distributed in a rural community in Northern Idaho. Survey links were provided to an administrator and supervising physician at the two local hospitals in this rural community to distribute to the healthcare providers (i.e., any individual who was providing direct care to patients regardless of credential) in those hospitals. The survey utilized the Nursing Community Apgar Questionnaire (NCAQ); however, the wording was modified to be inclusive of any type of healthcare provider. The survey consisted of five retention classes (i.e., *geographic* factors, *economic* factors, *scope of practice* factors, *medical support* factors, and *facility and community support* factors) with 10 factors per class. Each factor was assessed using two different Likert scales measuring the advantage/challenge an item had in retaining healthcare providers (-2 = major challenge, -1 = minor challenge, 1 = minor advantage, 2 = major advantage), and the importance of an item in the retention of healthcare providers (1 = very unimportant, 2 = unimportant, 3 = important, 4 = very important). Positive mean scores for the advantage scale indicated that respondents on average perceived those factors as advantageous while negative mean scores indicated respondents on average perceived those factors as challenges. For the importance scale, mean scores ≥ 3 indicated that respondents on average perceived those factors as important while mean scores < 3 indicated that respondents on average perceived those factors as unimportant. Classes and factors were compared to determine the highest rated for importance, advantage, and Agar score (importance*advantage). Participants were provided with a $25 Amazon gift card for participation and were given an opportunity to receive a second $25 Amazon gift card if they chose to participate in a follow-up interview. Interview data was not included in this study. This study was certified exempt by the University of Idaho Institutional Review Board.

### Data analysis

The Qualtrics survey platform was used for data collection and storage of survey responses (Qualtrics, Provo, UT). Data analysis was performed using IBM SPSS Statistics 25 (IBM Corp. Armonk, NY) and SAS version 9.4 (SAS Institute, Cary NC). Continuous variables were presented using means and standard deviations, while frequencies and percentages were used to describe categorical variables. Likert scale retention factors were presented and analyzed based on the methodology put forth by the developers of the NCAQ [29]. Retention factors were analyzed on both their importance and whether the factor was considered an advantage or challenge to retention. Importance scores were multiplied by advantage/challenge scores to create the weighted ‘Apgar’ score (Importance x Advantage = Apgar). The range of potential Apgar scores was from -8 to 8.

### Ethics approval

The project was certified exempt by the University of Idaho Institutional Review Board (Protocol: 21–162). All participants provided informed consent prior to completing the survey.

## Results

### Patient characteristics

Our sample included 30 respondents who completed the retention survey. Descriptive characteristics of survey respondents are presented in Table 1. Respondents were on average 44 years of age (SD = 12) and had 14 years of clinical practice experience (SD = 12). The highest proportions of respondents were male (50%), white (93%), and were physicians (52%).

**Table 1:** Descriptive statistics of survey respondents

Characteristic	M (SD)	
Age	44 (12)	
Years of clinical practice	14 (12)	
	**N (%)**	
Sex		
Male	15 (50.0)	
Female	13 (43.3)	
Prefer not to answer	2 (6.7)	
Ethnicity		
White	28 (93.3)	
Prefer not to answer	2 (6.7)	
Childhood Geographical Location		
Large city (500,000 or more)	6 (20.0)	
Suburb of a large city	2 (6.7)	
City of a moderate size (50,000 to 500,000)	3 (10.0)	
Suburb of a moderate size city	1 (3.3)	
Small city (10,000 to 50,000– other than a suburb)	6 (20.0)	
Town (2,500 to 10,000– other than a suburb)	6 (20.0)	
Small town (population less than 2,500)	6 (20.0)	
Profession		
Physician	15 (51.7)	
Physician Assistant	3 (10.4)	
Nurse	4 (13.8)	
Other	7 (24.1)	

### Advantages and challenges

Overall mean advantage/challenge scores are presented in Table 2. *Facility and community support* was identified as the highest advantage class influencing retention, with the highest mean advantage score of 2.0 (range of possible scores: -2 to 2). Across all classes, the top 10 factors identified as advantages to healthcare provider retention were CME benefit, teaching, perception of quality, obstetrics: prenatal care, obstetrics: deliveries/C-section, minor trauma (casting/suturing), emergency/stabilization care, inpatient care, recreational opportunities, and medical reference resources. The top ten challenges to retention were housing (availability and/or affordability), shopping and other services, demographics: underserved/pay or mix, electronic medical records, access to larger community, televideo support, social networking, spousal satisfaction (education, work, general), specialist availability, and nursing workforce.

**Table 2:** Overall mean scores for importance, advantages/challenges, and community Apgar scores

	Advantage or Challenge^a^	Importance^b^	Apgar Score^c^
**Economic class**			
Part-time Opportunities	-0.27	2.7	-0.73
Loan Repayment	0.20	3.1	0.62
Salary (Amount)	0.17	3.4	0.58
Signing Bonus/Moving Expenses	0.34	3.0	1.0
Length of Contract Flexibility	0.21	2.9	0.62
Perceived Fiscal Stability	0.17	3.3	0.56
Production Incentive	0.20	3.1	0.61
Retirement Package	0.37	3.2	1.2
CME Benefit	0.63	3.1	2.0
Competition	0.10	2.7	0.27
**Facility and community support class**			
Physical plant and equipment	0.13	3.2	0.42
Plans for capital investment	0.10	3.1	0.31
Electronic medical records	-0.77	3.4	-2.6
Leadership	0.40	3.4	1.4
Televideo support	-0.53	2.9	-1.6
Community need/support	0.53	3.2	1.7
Welcome and recruitment program	0.54	2.7	1.5
Medical reference resources	2.0	2.9	5.9
Delegated patient services	0.21	2.9	0.60
Moonlighting opportunities	0.27	2.6	0.69
**Geographic class**			
Access to larger community	-0.70	2.6	-1.8
Demographics: underserved/pay or mix	-0.77	2.8	-2.1
Housing (availability and/or affordability)	-1.2	3.2	-3.7
Schools	-0.25	3.2	-0.81
Social networking	-0.47	2.7	-1.3
Recreational opportunities	1.3	3.3	4.3
Spousal satisfaction (education, work, general)	-0.34	3.5	-1.2
Shopping and other services	-0.77	2.6	-2.0
Climate	0.60	2.8	1.7
Perception of Community	-0.10	2.8	-0.28
**Medical support class**			
Perception of quality	0.77	3.5	2.7
Stability of physician workforce	0.33	3.4	1.1
Specialist availability	-0.31	3.1	-0.95
Nursing workforce	-0.31	3.5	-1.1
Mid-level provider workforce	0.62	3.0	1.9
Ancillary staff workforce	0.30	3.1	0.92
Pharmacy services	0.31	3.1	0.97
Allied mental health workforce	-0.10	3.3	-0.33
Language support services	-0.24	2.8	-0.66
Call/practice coverage	0.03	3.5	0.10
**Scope of practice class**			
Obstetrics: prenatal care	0.79	3.1	2.5
Obstetrics: deliveries/C-section	0.79	3.1	2.5
Inpatient care	0.87	3.3	2.9
Emergency/stabilization care	0.86	3.3	2.9
Minor trauma (casting/suturing)	0.86	3.1	2.7
Office GYN procedures	0.61	2.9	1.8
Mental health	-0.17	3.2	-0.55
Mid-level supervision	0.30	2.8	0.84
Teaching	0.76	3.1	2.4
Administration	0.23	3.1	0.71

^a^Mean scores were calculated based on a -2 to 2 scale where -2 = major challenge, -1 = minor challenge, 1 = minor advantage, and 2 = major advantage

^b^Mean scores were calculated based on a 1 to 4 scale where 1 = very unimportant, 2 = unimportant, 3 = important, and 4 = very important

^c^Apgar scores were calculated by multiplying importance scores by advantage/challenge scores to create the weighted ‘Apgar’ score (Importance x Advantage/Challenge = Apgar)

### Importance

Overall mean scores for importance of provider retention are presented in Table 2. Medical support was identified as the most important class with 9 out of 10 factors above 3.0. Across all classes, the top ten factors of importance were perception of quality, salary (amount), spousal satisfaction (education, work, general), emergency/stabilization care, stability of workforce, leadership, call/practice coverage, nursing workforce, inpatient care, and electronic medical records. The bottom ten factors for importance (least important) were moonlighting opportunities, perception of community, climate, part-time opportunities, demographics: underserved/pay or mix, social networking, competition, access to larger community, shopping and other services, and welcome and recruitment program.

### Overall Apgar scores

Overall mean Apgar scores are presented in Table 2. The *facility and community support* class had the most impactful Apgar score which ranged from -2.6 to 5.9. Across all classes, the top 10 factors for the community Apgar scores (most important advantages) were minor trauma (casting/suturing), CME benefit, teaching, obstetrics: prenatal care, obstetrics: deliveries/C-section, perception of quality, inpatient care, emergency/stabilization care, recreational opportunities, and medical reference resources. The bottom 10 factors for the community Apgar scores (most important challenges) were electronic medical records, nursing workforce, specialist availability, access to larger community, spousal satisfaction (education, work, general), housing (availability and/or affordability), televideo support, shopping and other services, social networking, and demographics: underserved/pay or mix.

## Discussion

As a rural and frontier state, Idaho faces shortages of healthcare providers which in turn limits access to care for Idaho residents [7]. Multiple factors have been identified as potential barriers or facilitators to retention of healthcare providers in rural areas. However, few studies have focused on the U.S. population and, due to variation in characteristics of communities, it is important to explore community-level data to better understand how these factors affect retention in rural areas. Therefore, the purpose of this study was to explore perceived factors influencing healthcare provider retention in a rural community in Northern Idaho. A survey was administered to 30 healthcare professionals, seeking insights on primary factors affecting the retention of providers in rural areas. We observed that respondents identified the medical support class (e.g., nursing workforce) as the most pivotal element contributing to provider retention. However, *facility and community* support was the highest advantage class and had the highest Apgar scores, indicating it contained factors that healthcare providers identified as the most important advantages.

Previous studies have identified *economic* factors as important to provider retention in rural areas. A study conducted in Canada reported that income was an important factor regarding whether or not physicians would choose to practice medicine in a rural area [26]. In the current study, “CME benefits”, from the *economic* class, was considered to be one of the most important advantages to retention.

In a study conducted on family and community specialist physicians, rural physicians reported use of telemedicine more frequently than urban physicians; higher telemedicine use was associated with a higher reported value of telemedicine to support healthcare in the community [18]. In the current study, televideo support was observed as an important challenge to provider retention in rural areas which may be exacerbated by the higher rate of use of telemedicine among rural physicians [18]. Additionally, Prengaman et al. utilized the NCAQ on an Australian sample and reported that “availability of necessary materials and equipment” was one of the most important advantages in their sample [19]. This is consistent with our finding that medical reference resources was one of the most important advantages reported from our sample. Similarly, both Prengaman et al. and our study identified electronic medical records as an important challenge to retention [19]. 

Prior research has also identified the importance of *geographic* factors regarding retention of rural providers [19]. The nurses in Prengman et al.’s study reported that access to a large community and spousal support were both important challenges to retention of rural providers [19]. Furthermore, a study conducted in the U.S. utilizing the NCAQ to identify factors influencing medical students choosing to practice in rural areas reported that spousal satisfaction was one of the top reasons participants elected to practice medicine in rural areas [30]. In the current study, access to the larger community and spousal satisfaction were observed as important challenges to retention. Such differences in findings between studies conducted on U.S. samples may be indicative of regional variations in medical practice and/or local culture which further emphasizes the need for additional community-level research regarding rural retention of healthcare providers.

Previous studies have reported medical support factors as important to healthcare provider retention in rural communities [20]. Researchers using the NCAQ in a sample of rural north-eastern Australian healthcare providers reported that the nursing workforce was one of the most important advantages for retention of rural providers [20]. However, our study identified the nursing workforce as one of the most important challenges to retention. The nursing workforce was perceived as important in both studies but was perceived as an advantage in prior research and as a challenge in our study which may indicate differences in the sufficiency of the current nursing workforce between the samples. For example, Idaho has a significant healthcare provider workforce shortage, specifically related to the nursing profession [31]. The state of Idaho is in a nursing deficit of an estimated 1,119 nurses based on a comparison of Idaho with the U.S. national standard [31]. Additionally, emergency care, which is part of the *scope of practice* class, was reported as a challenge to retention in prior research, despite not being rated as a top factor [20]. In contrast, emergency/stabilization care in the current study was identified as a top advantage. These differences in findings between the U.S. and Australian studies might be attributed to variations in medical practice or structural differences in healthcare between the two countries [32]. 

### Limitations and future research

This study does have limitations. To begin, the results may not be representative of other rural regions of Idaho or across the U.S. Our sample size was limited by the population of the target region and the difficulty of recruiting participants from such a rural and remote area. Our study also may have potentially been influenced by social desirability bias due to the self-report nature of the survey instrument; however, the survey content was not of a stigmatizing or sensitive nature [33]. We are also limited in our ability to understand how nonparticipation may have affected which factors were reported as important advantages or challenges by providers; those who elected to not take the survey may have responded differently than those who elected to take the survey. There was also a limitation regarding the categorization of our question asking respondents to report what their profession was. Approximately one-fourth (24%) responded that their profession was “other” indicating that their profession was not listed as a response in the question; this limits our knowledge about whom our findings can be generalized.

## Conclusion

Our study identified several factors assessed by respondents as important advantages or disadvantages for healthcare provider retention in a rural and frontier community in northern Idaho. Generally, respondents reported multiple *economic*, *facility and community support*, *geographic*, and *medical support* factors as potentially important advantages and challenges to retention of providers in rural areas. Rural healthcare organizations looking to increase retention of their providers should give thought to targeting such factors due to their perceived importance to healthcare providers. Due to few prior studies on having been conducted on U.S. samples, and limited sample of this specific study, it is necessary for future research to be conducted on other rural samples across the U.S. to make comparisons of findings.

## Data Availability

The datasets used and analyzed during the current study are available from the corresponding author on reasonable request.
